# EPIGIST: An observational real-life study on patients with metastatic gastrointestinal stromal tumors receiving imatinib

**DOI:** 10.1371/journal.pone.0204117

**Published:** 2018-09-18

**Authors:** Olivier Bouché, Axel Le Cesne, Maria Rios, Loic Chaigneau, Antoine Italiano, Florence Duffaud, Thierry Lecomte, Dominique Arsène, Sylvain Manfredi, Thomas Aparicio, Stéphane Remy, Nicolas Isambert, Olivier Collard, Frank Priou, François Bertucci, Roland Sambuc, Ségolene Bisot-Locard, Olivier Bourges, Sylvie Chabaud, Jean-Yves Blay

**Affiliations:** 1 Department of Digestive Oncology, University Hospital, Reims, France; 2 Department of Cancer Medicine, Institut Gustave Roussy, Villejuif, France; 3 Department of Medical Oncology, Institut de Cancérologie de Lorraine—Alexis Vautrin, Nancy, France; 4 Department of Medical Oncology, Centre J. Minjoz Universitary Hospital Center, Besançon, France; 5 Department of Medical Oncology, Institut Bergonié, Bordeaux, France; 6 Department of Medical Oncology, La Timone University Hospital, Marseille and Aix -Marseille University (AMU), Marseille, France; 7 Department of Hepato-Gastroenterology and Digestive Oncology, University Hospital Trousseau, Tours, France; 8 Department of Hepato-Gastroenterology and Digestive Oncology, University Hospital Caen Normandy, Caen, France; 9 Department of Gastroenterology Oncology, University Hospital Dijon, Dijon, France; 10 Department of Medical Oncology, University Hospital Saint Louis, Paris, France; 11 Department of Medical Oncology, Clinique Paulmy, Bayonne, France; 12 Department of Medical Oncology, Centre Georges-François Leclerc, Dijon, France; 13 Department of Medical Oncology, Institut de Cancérologie Lucien Neuwirth, Saint-Priest-en-Jarez, France; 14 Department of Medical Oncology, Les Oudairies hospital CHD Vendée, La Roche sur Yon, France; 15 Department of Medical Oncology, Institut Paoli-Calmettes, Marseille, France; 16 Department of Public Health, La Conception Hospital, Marseille, France; 17 Oncology Business Unit, Novartis Pharma S.A.S., Rueil-Malmaison, France; 18 Biostatistics Unit, Centre Léon Bérard, Lyon, France; 19 Department of Medical Oncology, Centre Léon Bérard, Lyon, France; Istituto di Ricovero e Cura a Carattere Scientifico Centro di Riferimento Oncologico della Basilicata, ITALY

## Abstract

**Background:**

Gastrointestinal stromal tumors (GISTs) are rare, but represent the most common mesenchymal neoplasms of the gastrointestinal tract. EPIdemiology GIST, is an observational multicenter longitudinal follow-up cohort study reporting the prescribing patterns of imatinib in patients with GIST and the impact of the treatment in a real-world (standard clinical) setting.

**Methods:**

Eligible patients had a confirmed diagnosis of unresectable or metastatic *KIT*-positive GIST and started treatment with imatinib for the first time between May 24, 2002, and June 30, 2010. During routine visits, annual collection of clinical characteristics was requested, i.e., age, GIST stage at diagnosis, history, imatinib treatment duration and dosage, adherence, and concomitant medications. Survival outcomes were estimated using the Kaplan-Meier method. Other data were analyzed using descriptive statistics.

**Results:**

Of 151 patients enrolled, imatinib was initiated for 126 patients before enrollment and for 25 patients on the day of enrollment or soon after. The patient characteristics were similar to those in published prospective trials. The estimated 1-, 2-, 3-, and 4-year overall survival rates were 90.4% (95% confidence interval [CI; 84.8%-94.0%]), 84.7% (95% CI [78.1%-89.4%]), 73.0% (95% CI [65.0%-79.4%]), and 60.7% (95% CI [51.4%-68.8%]), respectively. The most common adverse events (AEs) were diarrhea (39%), asthenia (39%), eyelid or periorbital edema (32%), abdominal pain (23%), and anemia (21%). Eight of 126 serious AEs were possibly related to the treatment as assessed by investigators.

**Conclusions:**

Study results showed that patients in real-life populations are generally treated in accordance with national and international clinical recommendations and have outcomes comparable to those of patients in clinical trials.

## Introduction

Gastrointestinal stromal tumors (GISTs) are rare mesenchymal tumors of the gastrointestinal tract. The estimated incidence in Europe is between 6.6 and 14.5 cases per million persons per year [1–8]. GISTs usually occur in older adults with a median patient age of approximately 50 years, but GIST can be diagnosed at all ages [9].

GIST results from constitutively activated transmembrane receptor tyrosine kinase *KIT*, and more rarely of platelet-derived growth factor receptor alpha (*PDGFR*-α) in the cells of Cajal. The deregulation of *KIT* leads to a downstream cascade of intracellular signals, which leads to uncontrolled cell proliferation [10]. *KIT* (also known as CD117) expression is detectable using immunohistochemistry in most cases (95%) of GIST [11].

Surgical resection is the standard of care for localized, primary GISTs [9]. Until the availability of imatinib, unresectable or metastatic GISTs had a poor prognosis because they are highly resistant to cytotoxic chemotherapy. In the pre-imatinib era, the median overall survival (OS) was 19 months for patients with metastatic GIST, 9 months for patients with local and metastatic recurrence, and 12 months for patients with local recurrence [11].

The prognosis for these patients has improved dramatically since the introduction of imatinib, a drug that inhibits the tyrosine kinase activity of several receptors including *KIT* and *PDGFR-*α. Imatinib was approved by the US Food and Drug Administration (FDA) and the European Medicines Agency (EMA) in 2002 for the treatment of *KIT*-positive unresectable and/or metastatic malignant GIST at a starting dose of 400 mg/day [12]. The activity of imatinib has been well documented in clinical studies in the early 2000 [11, 13, 14]. Currently, there are no data with respect to results in the real-world setting.

EPIdemiology GIST (EPIGIST) was required by the French health authorities in order to confirm that the clinical trial results could be transposed in real-world settings [15]. This real-life observational study aimed to describe the profile of patients with GIST receiving imatinib to assess the real-world imatinib prescribing patterns, and the safety and efficacy in the health of the treated population. Information about the use of health care resources by patients, the economic impact of their clinical management, and their quality of life (QoL) was also recorded.

## Methods

### Study design

EPIGIST was an observational, multicenter, longitudinal, follow-up, cohort study mandated by the French Health Authorities and conducted in metropolitan France. A total of 1691 concerned specialists (private and public, oncologists, gastroenterologists, and internists) in Metropolitan France were eligible. The investigators were randomly selected, stratified by their geographical location and practice type to minimize bias, and to be representative of the physicians who take care of GIST patients. All the physicians were contacted through letters, and those who agreed to participate in the study were considered. This study was complied with ethical principles of the Declaration of Helsinki, Good Clinical Practice, and applicable local regulations. Written and dated informed consent was obtained from each patient. The study was approved by French Health Comité Consultatif sur le Traitement de l'Information en Matière de Recherche dans le Domaine de la Santé (CCTIRS, the Advisory Committee on Information Processing in Material Research in the Field of Health) and Commission nationale de l'informatique et des libertés (CNIL, the French Data Protection Authority).

### Patient population

Eligible patients were adults diagnosed with an unresectable or metastatic *KIT*-positive GIST treated with imatinib for the first time between its market introduction on May 24, 2002, and June 30, 2010. Patients, who were not residents of France or who were already enrolled in a therapeutic study with imatinib were not included, except patients enrolled in the BFR-14 trial. Patients from BFR-14 trial were included in the EPIGIST study as the first part of the BFR-14 study (before randomization) was in line with the prescription pattern of imatinib for all patients [16].

The cohort was established with the patients who were already undergoing imatinib treatment (initiated between its market introduction and the start of enrollment period in December 2006), defined as prevalent patients, and was completed with those patients who were initiated on imatinib treatment at the time of enrollment (incident patients). This cohort was followed up for at least 2 years.

To correct the overestimation of the OS owing to the inclusion of patients who were prevalent but by definition alive at the time of inclusion in the study, a registry of patients under imatinib who died during the study eligibility period was used.

### Objectives

Primary objectives were to describe the profile of treated patients, the prescription patterns (indication, dosage, concomitant drug, etc.), and the impact of the treatment on population health (morbidity, mortality, and tolerability). Secondary objectives were to describe the socio-economic impact of the disease, patients’ QoL, and to compare the real-world setting data to the clinical trial data.

### Data collection

Patient characteristics (sex, age) and clinical characteristics (age and GIST stage at diagnosis, history, imatinib treatment duration and dosage, adherence, concomitant medications) were collected at baseline. Annual collection of clinical characteristics (imatinib treatment duration and dosage, adherence, and concomitant medications) during a routine visit was requested. All serious and non-serious adverse events were collated to report the safety and tolerability of imatinib. The patients’ QoL was assessed using the SF-36 and QLQ-C30 questionnaires [17, 18]. Finally, a self-administered questionnaire was completed by the patients every 6 months to report the health care consumption and any sick leave taken.

To evaluate the survival rate on imatinib, a registry was set up, including all the patients who died during the period corresponding to market introduction of the product and the start of the enrollment period.

### Statistical analyses

Descriptive statistics was performed for quantitative variables (mean, standard deviation, median, confidence interval, and extreme values). Qualitative variables were described in terms of size and frequency. Population analysis includes prevalent and incident patients. Survival analysis also accounts for death registry patients to correct the overestimation caused by inclusion of prevalent alive patients only. The analysis of OS was performed using the Kaplan-Meier method, taking into account the date of imatinib initiation, the date of death, or date of last known contact. Median follow-up was estimated using the reverse Kaplan-Meier method.

## Results

Of the 1691 physicians, 407 were randomly selected and contacted. Two hundred ninety-three (72%) responded and 52 (13%) agreed to participate in the study, of which 4 physicians gave their consent later. Thirty-one investigators enrolled at least 1 patient. The main reasons given by the physicians who declined to participate were not treating any patients with GIST at the time of study (70%), lack of time availability (11%), and participation in another study (6%).

### Demographics and disposition

Between December 2006 and June 2010, 164 patients were enrolled by 31 investigators. Each recruiting investigator included an average of 5.3 patients (range, 1-25 patients). Of 164 patients who were initially included, 13 were withdrawn from analysis due to major protocol violations. Those 13 patients were all in the randomized part (continue imatinib vs stop imatinib) of BFR-14 study, which would have been a major influence of the treatment. Hence, per protocol cohort consisted of 151 patients. There were minor protocol violations for 16 patients: 8 treated for a resectable or resected GIST, 1 stopped imatinib before inclusion, and 7 started imatinib after inclusion. There were 126 prevalent and 25 incident patients.

The median time from diagnosis to enrollment was 21.8 months (range, 0.6-138.9). The median follow-up since enrollment was 2.7 years (83% of patients were followed up for at least 12 months, 68% for 24 months, 26% for 36 months, and 5.5% for up to 48 months or longer).

### Clinical characteristics at diagnosis and enrollment

The study population was predominantly male (58.3%). Median age at diagnosis was 60.0 years (range, 21.0–86.0 years). Most of the patients had localized disease at the time of diagnosis (53%). There were 123 patients (96.1%) with Eastern Cooperative Oncology Group (ECOG) performance status (PS) of 0 or 1, and 5 (3.9%) patients with PS of 2 or 3 at the enrollment. The previous treatment performed for GIST was mainly surgery (111 patients). Two patients had previously undergone radiotherapy, and 1 previously received chemotherapy. The locations of primary tumors are listed in Table 1.

**Table 1:** Location of the primary tumor at diagnosis.

	Metastatic n = 66	Non-metastatic n = 85	Total N = 151
**Location of the tumor, n (%)**			
Esophagus	2 (3.0%)	0 (0.0%)	2 (1.3%)
Stomach	32 (48.5%)	38 (44.7%)	70 (46.4%)
Colon	1 (1.5%)	1 (1.2%)	2 (1.3%)
Rectum	0 (0.0%)	8 (9.4%)	8 (5.3%)
Mesentery	2 (3.0%)	3 (3.5%)	5 (3.3%)
Colon + stomach	1 (1.5%)	1 (1.2%)	2 (1.3%)
Other	5 (7.6%)	2 (2.4%)	7 (4.6%)
Small intestine	23 (35%)	32 (37.6%)	55 (36.5%)
Duodenum	5 (7.6%)	8 (9.4%)	13 (8.6%)
Jejunum	4 (6.1%)	5 (5.9%)	9 (6.0%)
Ileum	4 (6.1%)	7 (8.2%)	11 (7.3%)
Small intestine, not otherwise specified	10 (15.2%)	12 (14.1%)	22 (14.6%)

### Clinical characteristics at the time of imatinib initiation

The clinical characteristics of patients on initiation of imatinib are listed in Table 2. Most patients had metastatic disease at the time of imatinib initiation. The 2 main sites for the metastases were the liver (72.5%) and the peritoneum (41.8%). Eighty-five patients (56.3%) had localized disease, and 66 (43%) were metastatic at initial diagnosis. For the primary tumor, the distribution according to National Institutes of Health (NIH) risk classification [16] was as follows: high risk, 68 (80.0%); intermediate risk, 6 (7.1%); low risk, 1 (1.2%); and unknown, 10 (11.8%). According to the Armed Forces Institute of Pathology (AFIP) [17], the classification of the primary was as follows: high risk, 42 (49.4%); intermediate risk, 20 (23.6%); low risk, 5 (5.9%); very low, 2 (2.4%); and unknown, 15 (17.6%).

**Table 2:** Clinical characteristics on initiation of imatinib.

	Metastatic diagnosis	Non-metastatic diagnosis	Total
	n = 66	n = 85	N = 151
GIST staging, n (%)			
Localized	0 (0.0%)	7 (8.2%)	7 (4.6%)
Localized non resectable	0 (0.0%)	16 (18.8%)	16 (10.6%)
Metastatic	66 (100.0%)	60 (70.6%)	126 (83.4%)
Resected without metastasis	0 (0.0%)	2 (2.4%)	2 (1.3%)
Location of metastases, n (%)			
Non-metastatic initiation imatinib	0 (0.0%)	25	25
Missing data	1	1	2
Peritoneal	10 (15.4%)	12 (20.3%)	22 (17.7%)
Hepatic	36 (55.4%)	22 (37.3%)	58 (46.8%)
Other	3 (4.6%)	5 (8.5%)	8 (6.5%)
Peritoneal + hepatic	11 (16.9%)	11 (18.6%)	22 (17.7%)
Hepatic + peritoneal + other	2 (3.1%)	2 (3.4%)	4 (3.2%)
Peritoneal + other	1 (1.5%)	3 (5.1%)	4 (3.2%)
Hepatic + other	2 (3.1%)	4 (6.8%)	6 (4.8%)

The median time from diagnosis to imatinib initiation was 2.9 months (range, 0.0–135.1) overall, 1.3 months (range, 0.0–26.0) for metastatic patients at diagnosis, and 13.0 months (range, 0.0–135.1) for non-metastatic patients at diagnosis.

Overall, 98% of patients received imatinib at a starting dose of 400 mg/day as recommended in the summary of product characteristics. The dose had to be reduced for 18% of patients. The dose was increased for 31 (20.6%) patients to up to 800 mg/day for 27 (17.9%) patients on an average of 29.8 months (range, 4.3–65.9) after initiation of imatinib. Eighty-four patients (56.0%) had no treatment interruption. The median duration of their treatment was 42.6 months (range, 4.9–86.7). Sixty-two patients (41.9%) interrupted imatinib therapy at least once, and 15 resumed therapy subsequently. There is no information on continuation or interruption for 5 patients. The reasons for interruption were disease progression (66.2%), side effects (16.9%), and others (15.5%), including disease control (4.2%). At baseline, among 126 prevalent patients (previously treated), 98.4% were taking at least 90% of the prescribed dose, according to the investigator. Adherence (evaluated by the investigator) remained at this high level after 1 year (94.0% of 122 patients) and 2 years (93.5% of 99 patients).

### Efficacy

**Fig 1:**
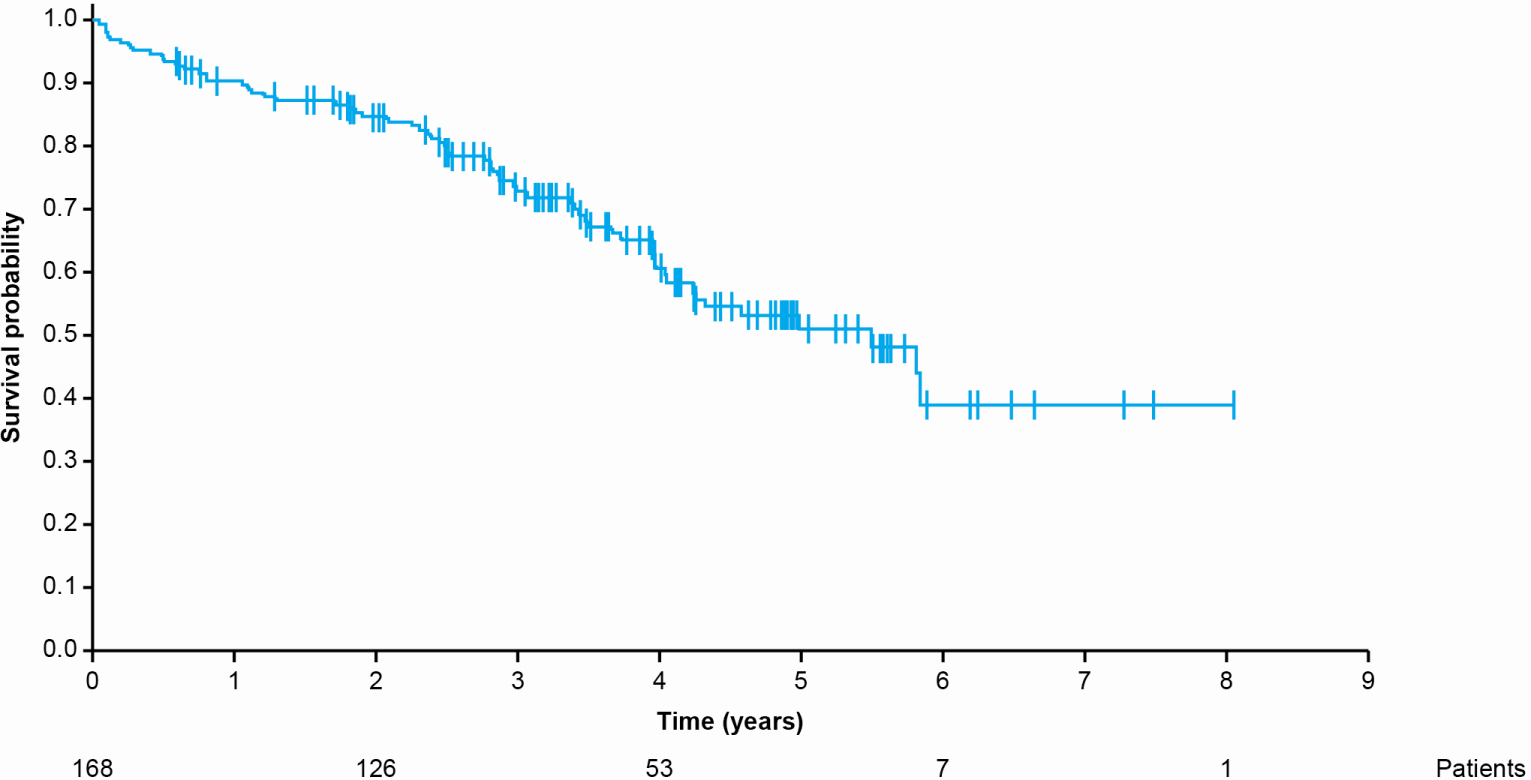
Overall survival from the date of imatinib initiation.

The OS analysis was conducted in 168 patients (151 enrolled patients and 17 patients from the registry of deaths) from the date of imatinib initiation to the date of death or date of last known contact, including patients who had interrupted or discontinued imatinib therapy. Sixty-three deaths were reported. The median follow-up since initiation of imatinib was 4 years (range, 0.6-8.0). The estimated 1-, 2-, 3-, and 4-year OS rates were 90.4% (95% CI [84.8%-94.0%]), 84.7% (95% CI [78.1%-89.4%]), 73.0% (95% CI [65.0%-79.4%]), and 60.7% (95% CI [51.4%-68.8%]), respectively (Fig 1).

### Safety

A total of 1302 events (adverse events [AEs] and serious AEs [SAEs]) were reported involving 148 (98%) patients, regardless of whether they were related to the treatment (Table 3). The most common gastro-intestinal events were diarrhea (39%), abdominal pain (23%), and nausea (19%). The most common general disorders and administration site conditions were asthenia (39%), death (20%), and edema of lower limbs (14%). Eyelid or periorbital edema was reported in 32%, cramps in 19%, and anemia in 21% of patients.

**Table 3:** Adverse events (according to low-level term) reported in at least 10 patients during follow-up.

Low-level term	Patients n = 151 n (%)	Number of occurrences of the AE	AE frequency in the warnings and precautions section in the current label for EU
Diarrhea	59 (39.1%)	94	≥ 10%
Asthenia	59 (39.1%)	97	≥ 10% (fatigue)
Abdominal pain	34 (22.5%)	40	≥ 10%
Palpebral edema	34 (22.5%)	42	≥ 1 to < 10 (eyelid edema)
Anemia	32 (21.2%)	43	≥ 10%
Death NOS	30 (19.9%)	31	
Cramp	29 (19.2%)	42	≥ 10% (muscle spasms and cramps)
Nausea	28 (18.5%)	37	≥ 10%
Lower limb edema	21 (13.9%)	25	≥ 10% (fluid retention and edema)
Hospitalization	20 (13.2%)	27	
Impairment of general condition	17 (11.3%)	20	≥ 1 to < 10 (weakness)
Periorbital edema	14 (9.3%)	19	≥ 10%
Edema	12 (7.9%)	14	≥ 10% (fluid retention and edema)
Vomiting	11 (7.3%)	14	≥ 10%
Bowel motility disorder	10 (6.6%)	10	
Neutropenia	10 (6.6%)	14	≥ 10%

NOS, not otherwise specified

Of 126 SAEs reported (Table 4) involving 85 patients, 8 were possibly related to the treatment according to the investigators. SAEs related to imatinib include asthenia, liver progression, rectal hemorrhage, gastrointestinal perforation, interstitial pneumopathy, pulmonary edema, and rash. During the study period, 46 deaths were reported, most of which occurred in a context of disease progression; 1 death was suspected to be related to the treatment (patient with acute pulmonary edema and pulmonary embolism).

**Table 4:** Serious adverse events (MedDRA system organ classes) reported during follow-up.

MedDRA system-organ class	Total number of “serious” reports	Number of reports suspected of being related to the treatment by the investigator
General disorders and administration site conditions	24	1
Neoplasms benign and malignant	23	1
Gastrointestinal disorders	19	3
Respiratory and thoracic disorders	9	2
Cardiac disorders	7	0
Infections and infestations	7	0
Surgical and medical procedures	6	0
Blood disorders	7	0
Injury, poisoning	5	0
Nervous system disorders	4	0
Skin disorders	2	1
Investigations^a^	2	0
Psychiatric disorders	2	0
Reproductive system disorders	2	0
Renal and urinary disorders	1	0
Hepatobiliary disorders	1	0
Metabolism and nutrition disorders	2	0
Vascular disorders	1	0
Eye disorders	1	0
Endocrine disorders	1	0
**Total**	**126**	**8**

^a^ Investigations include biological examinations abnormalities, examinations (ECG, CT scan etc.) induced by abnormalities

### QoL

The QoL data using SF-36 and QLQ-C30 questionnaires [10, 14] were available for 110 patients at baseline, 92 patients after 6 months, 80 patients after 12 months, and 77 patients after 18 months.

The patients’ QoL remained generally stable during imatinib therapy, with slight improvement in some mean SF-36 physical score (PCS) and mental score (MCS) (Table 5).

**Table 5:** Evaluation of physical and mental scores, as measured by the SF-36, after 6 months, 12 months and 18 months follow-up in relation to the inclusion.

Relative evaluation (n = 133)		*P* value
After 6 months of follow-up	N = 70	
PCS	2.8 ± 19.8	0.241
MCS	-3.0 ± 20.4	0.225
After 12 months of follow-up	N = 65	
PCS	0.1 ± 20.0	0.978
MCS	1.7 ± 23.3	0.559
After 18 months of follow-up	N = 62	
PCS	2.9 ± 20.2	0.259
MCS	4.9 ± 26.8	0.157

PCS, physical score; MCS, mental score

For the QLQ-C30, after 6 months of follow-up, 28.8% of the patients showed improvement in the total score (≥10 points) of their QoL and 47.9% of patients remained stable (-10 to <10). After 12 months, 22.2% of patients had improved, 47.6% were stable; and after 18 months, 25.8% had improved, and 51.5% remained stable.

### Use of health care resources

Overall, 129 patients sent at least 1 self-administered questionnaire, corresponding to 6 months of follow-up. The mean follow-up per patient was 24 months.

Almost all of the patients (97.7%) consulted a physician at least once during the study period. On average, each patient attended 9.4 consultations per year of follow-up. Over the period studied, almost all (96.1%) of the patients consulted a GIST specialist. Precisely, 77.5% of patients were followed up by a general physician and a specialist.

Almost all patients (99.2%) underwent at least 1 investigation during the study period. On average, each patient underwent 11.4 investigations per year of follow-up. The most common investigations were computed tomography (CT) scans (94.6%) and blood tests (97.7%). Positron emission tomography (PET) and magnetic resonance imaging (MRI) scans were performed in 30.2% and 17.8% of cases, respectively.

Twenty-eight (21.7%) patients were hospitalized at least once. These patients had a mean of 0.98 hospitalizations per year. The mean number of hospitalization per patient per year was 0.22. Thirty-two (24.8%) patients had at least 1 period of sick leave. These patients took an average of 2.4 periods of sick leave per year, and a mean total of 135 days per year of follow-up per patient. The mean number of sick leaves per patient per year was 0.83. The mean duration of sick leave period was 47 days.

## Discussion

Before the imatinib era, there was no effective treatment for unresectable or metastatic GISTs. In 2001, Joensuu et al [19] reported a dramatic clinical response after 2 weeks of treatment with imatinib in a patient treated for a metastatic GIST. Imatinib immediately changed the clinical management and the prognosis of this tumor. Several prospective randomized studies enrolled patients with advanced GIST. The pivotal B2222 phase II study compared 400 to 600 mg of daily imatinib [20]. The EORTC 62005 [21] and S0033 [13] phase III studies compared 400 to 800 mg of daily imatinib. The data have additionally been explored in the gastrointestinal stromal tumor meta-analysis group (MetaGIST) project [14].

This real-life observational study of imatinib in patients with metastatic GISTs in France confirmed the results of previously reported prospective randomized trials. The characteristics of patients enrolled in this observational EPIGIST study were similar to those of patients in these prospective trials. The population was predominantly male, with GISTs most commonly located in the stomach and the small intestine. The median age at diagnosis was 60 years in EPIGIST and between 54 and 62 in these prospective trials.

Usually, the results of studies conducted in a real-life setting are less favorable compared to results of clinical trials in which possible sources of methodological bias are effectively controlled. A potential source of bias in this study could be that the investigators were selected by randomization in order to be representative of prescription patterns, but some of them did not agree to participate in the study. Besides, it could be hypothesized that the investigators selected their patients, and that their recruitment was not exhaustive or systematic.

In line with the prescribing information of imatinib, which is indicated for metastatic/advanced GIST, the vast majority of patients in EPIGIST had metastatic disease at the initiation of the treatment. Patients with a localized stage at treatment initiation showed recurrence of GIST in most cases. The initial dose of imatinib was the recommended dose of 400 mg per day. The use of imatinib in most of the patients was consistent with the framework provided by the regulatory approval. This study confirmed the positive impact of imatinib therapy on OS in patients with GIST. The AEs reported during this study reflect those reported in the clinical studies [11, 13, 14]. The frequency of majority of AEs reported in this study was similar to that reported in the label (summary of product characteristics [SPC]) [22]. But a few AEs were either not reported or not with a frequency as high as that reported in the label (SPC) like thrombocytopenia, headache, dyspepsia, dermatitis/eczema/rash, musculoskeletal pain including myalgia, arthralgia, bone pain, weight increased. There is a vast and long-standing experience with imatinib safety. Majority of the AEs reported in this study were previously known for imatinib. This observational study involving 151 patients is not powered to detect rare side effects, or to provide more details regarding the frequency of AEs. This study is a contribution to pharmacovigilance among thousands of other cases leading to better precision in defining the safety profile. Also, we believe it is not possible to design any recommendations from the information on AEs collected during this study.

The 4-year OS was 60.7% in EPIGIST, which is similar to or even greater than the OS reported in other studies. The median OS of the B2222 study and of the 400 mg arm of the MetaGIST study was 57 and 49 months, respectively [13, 14]. The patients in real-life populations have an outcome comparable to that of patients in clinical trials, confirming that imatinib provides a high level of activity in a general population.

The compliance reported by the investigators was high and was maintained throughout the study. However, a recent study showed that physicians, patients, and collaterals greatly overestimated patients’ adherence to treatment [23]. As in the EPIGIST study, compliance was assessed only by the investigators, hence we must consider that in reality it may be lower.

Patient monitoring was mainly performed by a GIST specialist. The CT scan and blood tests were the most common examinations. The data about the management of patients in real life matches the recommendations of the French guideline set out in the “Thesaurus National de Cancérologie Digestive” [24] as well as in the European Society for Medical Oncology (ESMO) Guidelines [9]. The diagnosis of GIST has a direct impact on the work productivity (in terms of economic wealth) of patients: 21.7% of patients were hospitalized at least once, and 24.8% had a sick leave during the period.

The scope of this study is limited as only descriptive statistics were included. Also, statistical analyses were not performed by subgroups, as any subgroups were not defined in the cohort a priori. As only 151 patients were included in the study, subgroup analyses would have very less power to show any statistical differences between groups, unless the difference was very high. Although there is an option of performing multivariate analysis of overall survival by adjusting the confounding factors, as this is a real-life study, a lot of confounding factors that were not necessarily collected may exist, and such analyses would remain biased and therefore were not considered.

## Conclusions

In conclusion, this study confirms the feasibility of imatinib treatment in routine practice. The reported results confirm those obtained in clinical studies for efficacy and safety in patients with metastatic GISTs. The prescriptions generally comply with the terms of the approved indication. After the initiation of this study, imatinib obtained approval for the adjuvant treatment of GIST in patients who underwent surgical resection. A recent study has shown that the adjuvant treatment of GIST with imatinib was cost-effective [25]. The present study provides novel information on compliance, health care management, and QoL of patients, which were not addressed in prospective clinical trials, and which will be useful for the delineation of future clinical trials in first line, with economic assessment.

### Earlier presentations

- Bouché, A Le Cesne, M Rios, L Chaigneau, B Bui Nguyen, X Parot, J-Y Blay. Les tumeurs stromales gastrointestinales avancées traitées par imatinib en France: quelle prise en charge dans la « vraie » vie? JFHOD 2012 (Journées Francophones d’Hépato-gastroentérologie et d’Oncologie Digestive). Abstract P2 [poster presentation].
- Bouché, A Le Cesne, M Rios, L Chaigneau, B Bui, X Parot, J-Y Blay. Real-life management of advanced gastrointestinal stromal tumors (GIST) treated with imatinib in France. ESMO 2012. Abstract 2620 [poster presentation].
- A Mathieu-Boue, O Bouché, A Cioffi, M Rios, F Duffaud, L Chaigneau, I Ray-Coquard, J-Y Blay, A Le Cesne, C Deschaseaux. Malignant gastrointestinal stromal tumors treated with imatinib in France: Results in unselected patients. ASCO 2009. Abstract e22177 [meeting abstract].
- Bouché, A Cioffi, M Rios, A Blesius, L Chaigneau, I Ray-Coquard, J-Y Blay, A Le Cesne, S Bisot-Locard, C Deschaseaux. Malignant gastrointestinal stromal tumors treated with imatinib in France: Results in unselected patients. ESMO 2009. Abstract 9415 [poster presentation].
- J-Y Blay, O Bouché, M Cucherat, M Goldberg, P Grosclaude, R Sambuc, S Bisot-Locard, C Deschaseaux, J Sailly. Malignant gastrointestinal stromal tumors treated with imatinib in France: Efficacy in real life. ISPOR 2009. Abstract PCN10 [poster presentation].

## Abbreviations

AE: adverse event
AFIP: Armed Forces Institute of Pathology
CT: computed tomography
CI: confidence interval
ECOG: Eastern Cooperative Oncology Group
EMA: European Medicines Agency
EPIGIST: EPIdemiology GIST
ESMO: European Society for Medical Oncology
FDA: US Food and Drug Administration
GIST: gastrointestinal stromal tumor
MCS: mental score
MetaGIST: gastrointestinal stromal tumor meta-analysis group
MRI: magnetic resonance imaging
NIH: National Institutes of Health
OS: overall survival
*PDGFR*-α: platelet-derived growth factor receptor alpha
PET: positron emission tomography
PCS: physical score
PS: performance status
SAEs: serious adverse events
QoL: quality of life
